# Re: *Insight into ECMO, mortality and ARDS: a nationwide analysis of 45,647 ECMO runs* (Friedrichson et al., *Critical Care*, January 2021)

**DOI:** 10.1186/s13054-021-03529-1

**Published:** 2021-03-26

**Authors:** Alex Warren, Luigi Camporota, Stephane Ledot, Ian Scott, Alain Vuylsteke

**Affiliations:** 1 Division of Anaesthesia, University of Cambridge, Cambridge, UK; 2 Department of Asthma, Allergy and Lung Biology, King’s College London, London, UK; 3 Guy’s & St Thomas’ Hospital, London, UK; 4 Royal Brompton & Harefield Hospitals, London, UK; 5 Aberdeen Royal Infirmary, Aberdeen, UK; 6 Royal Papworth Hospital, Papworth Road, Cambridge, CB2 0AY UK

**To the editor**

We read with interest the well-conducted analysis presented by Friedrichson et al. on the provision of ECMO in Germany from 2007 to 2018 [1].

The authors note that by 2018, there were 231 centres in Germany providing veno-venous ECMO support (VV-ECMO), with a median case volume of 4 patients per year; 70% of centres reported 10 or fewer cases annually. In the United Kingdom, since 2011, ECMO for respiratory failure has been provided by six centres in a hub-and-spoke network [2], similar to that the authors propose. We estimate from the data provided that in 2018, Germany had one VV-ECMO centre per 358,000 head of population; in the UK this figure was one per 11 million [3]. Our recent analysis of the first six years of this service demonstrated a median annual case volume of 37.5 patients per centre and overall ICU mortality of 26% [2].

There are likely multiple factors explaining the discrepancy between the UK data and the hospital mortality of 54% reported by Friedrichson et al. [1]. Firstly, even including neonatal and paediatric patients, 70% of patients receiving VV-ECMO in the German study were aged above 50; for the UK dataset, the corresponding figure was 33%. As the authors state, age is a key prognostic indicator for outcome [4]. The authors analysed patients who received VV-ECMO with or without ARDS, reporting essentially identical mortality, and conclude that some of these patients may not have had an indication for ECMO support. We would suggest that the underlying pathophysiology may be more important than simply meeting definition criteria of ARDS. In our study, patients with certain underlying pathologies, such as asthma, were more likely to survive [2].

Although a lower short-term mortality per se does not necessarily mean a ‘better’ system, the benefit of experienced multidisciplinary teams—which include nursing, perfusion and therapy staff—concentrated in higher volume ECMO centres, is in our view inarguable. This experience does not just improve care for the individual patient, but also informs on patient selection, which is key to driving better outcomes. Furthermore, a centralised network with a single governance structure provides further advantages on equity of access and sharing best practice. For the above reasons, we would entirely agree with the authors’ conclusions that ‘*treatment in specialised centres clearly results in reduced mortality*.’

Yours sincerely,

## Data Availability

None required.
