# Supplementary material for: Re: Insight into ECMO, mortality and ARDS: a nationwide analysis of 45,647 ECMO runs (Friedrichson et al., Critical Care, January 2021)
Source: Crit Care. 2021 Mar 26;25:123. doi: 10.1186/s13054-021-03529-1 (PMC7995399; doi:10.1186/s13054-021-03529-1)
Supplement: Related texts

# Reply: Authors' response

**Authors:** Benjamin Friedrichson, Haitham Mutlak, Kai Zacharowski, Florian Piekarski

**Affiliations:** 7 grid.411088.4 0000 0004 0578 8220 Department of Anaesthesiology, Intensive Care Medicine and Pain Therapy, University Hospital Frankfurt, Goethe University, Theodor-Stern-Kai 7, 60590 Frankfurt am Main, Germany; 8 grid.419837.0 Department of Anaesthesiology, Intensive Care Medicine and Pain Therapy, SANA Klinikum Offenbach, Starkenburgring 66, 63069 Offenbach am Main, Germany

To the editor

We would like to thank Drs. Warren et al. for their valuable letter, which underlines the impact of our recently published manuscript regarding ECMO therapy in Germany.

Data and especially the clinical setting published by Warren et al. are indeed completely contrary to the situation in Germany [5]. In addition to the lower number of centres in England compared to Germany, there is also a significantly lower ECMO incidence rate of approx. 3.05 per 1 million population compared to 18.28 per 1 million population [6]. Whether this clear difference is solely due to a different indication, e.g. inclusion of older patients, remains to be answered by further studies.

We agree with Warren et al. that a consideration of the underlying pathophysiology in acute respiratory syndrome (ARDS) makes an important difference in therapy and prognosis. Especially the mortality rates in H1N1 patients treated with ECMO have been shown to be lower than in other ARDS patients treated with ECMO [7]. The clinical entity of ARDS has multiple causes and is therefore particularly difficult to address in guidelines [8]. Currently, the final stage of the disease, i.e. refractory hypoxaemia, and its graduation, as well as the clinical course are the basic parameters used for ECMO indication. For this very reason, we are convinced that a centre formation with the experience, not only with regard to the interprofessional team, but also with regard to the underlying pathophysiology, can lead to a significant reduction in mortality. Analysing the underlying disease of ARDS is an important aspect; however, this was not possible due to the available data set of the Federal Statistical Office. We are convinced that a national registry for all ECLS treatments, as established in the United Kingdom, would be desirable for Germany. With the introduction of the German Interdisciplinary Association for Intensive and Emergency Medicine (DIVI) ECMO Covid-19 registry, a first step in the right direction has been taken [9].

Yours sincerely,
